# Information in small neuronal ensemble activity in the hippocampal CA1 during delayed non-matching to sample performance in rats

**DOI:** 10.1186/1471-2202-10-115

**Published:** 2009-09-15

**Authors:** Susumu Takahashi, Yoshio Sakurai

**Affiliations:** 1 Khoyama Center for Neuroscience, Faculty of Computer Science and Engineering, Kyoto Sangyo University, Kyoto 603-8555, Japan; 2 Precursory Research for Embryonic Science and Technology (PRESTO), Japan Science and Technology Agency, Kawaguchi 332-0012, Japan; 3 Department of Psychology, Kyoto University, Kyoto 606-8501, Japan; 4 Core Research for Evolution Science and Technology (CREST), Japan Science and Technology Agency, Kawaguchi 332-0012, Japan

## Abstract

**Background:**

The matrix-like organization of the hippocampus, with its several inputs and outputs, has given rise to several theories related to hippocampal information processing. Single-cell electrophysiological studies and studies of lesions or genetically altered animals using recognition memory tasks such as delayed non-matching-to-sample (DNMS) tasks support the theories. However, a complete understanding of hippocampal function necessitates knowledge of the encoding of information by multiple neurons in a single trial. The role of neuronal ensembles in the hippocampal CA1 for a DNMS task was assessed quantitatively in this study using multi-neuronal recordings and an artificial neural network classifier as a decoder.

**Results:**

The activity of small neuronal ensembles (6-18 cells) over brief time intervals (2-50 ms) contains accurate information specifically related to the matching/non-matching of continuously presented stimuli (stimulus comparison). The accuracy of the combination of neurons pooled over all the ensembles was markedly lower than those of the ensembles over all examined time intervals.

**Conclusion:**

The results show that the spatiotemporal patterns of spiking activity among cells in the small neuronal ensemble contain much information that is specifically useful for the stimulus comparison. Small neuronal networks in the hippocampal CA1 might therefore act as a comparator during recognition memory tasks.

## Background

Hippocampal formation has been identified as an important substrate for declarative memory for a broad range of materials in humans [1,2]. In contrast, in rodent studies, two views respectively hold that the hippocampus is dedicated to spatial memory processing [3] and that it associates general memory items [4]. Regarding the spatial view, results of several analyses of the stability of place cells have shown that pattern separation and pattern completion are apparent in neuronal ensembles of the hippocampus [5,6]. In support of the general view, several experiments directly showed activity related to match/non-match conditions using recognition memory tasks, such as a delayed non-matching-to-sample (DNMS) task [7-10]. Spatial information can be regarded as multiple items that are mutually associated according to temporal relations [11]. Therefore, place cells might code multiple events constructing a place experienced in the past [4]. From this viewpoint, the lines of evidence for the general and spatial views are not contradictory and are consistent with the view of the hippocampus as being capable of auto-associative functions to retrieve entire episodes [12]. On the other hand, the matrix-like organization of the hippocampus with several inputs and outputs has inspired some researchers to propose the hippocampal comparator theory [13-15], which suggests that the hippocampus supports comparison, which might be one element in the match/non-match judgements. In this context, several lines of evidence obtained from analyses of single neurons in spatial and non-spatial behavioral tasks suggest that the hippocampus is critical for episodic-like representations. Nevertheless, our knowledge related to neuronal computations in the working brain of behaving animals is limited; most of it has been inferred exclusively from changes in the firing rates of individual cells accumulated through many trials [16,17]. Therefore, to understand the hippocampal function completely, the actual encoding of information by the hippocampal neuronal networks of multiple neurons in a single trial during memory tasks must be elucidated. Recently, some researchers have attempted to elucidate the functioning of neuronal networks of the cerebral cortex using an artificial neuronal network classifier as a decoder that enables us to analyze spatiotemporal firing patterns among all observed cells in a single trial [18-20]. Consequently, using multi-neuronal recording and an artificial neural network classifier as a decoder, we analyzed spatiotemporal firing patterns among cells in the hippocampal CA1 of rats. We report its neuronal ensemble code in a single trial of a DNMS task.

## Results

**Figure 1:**
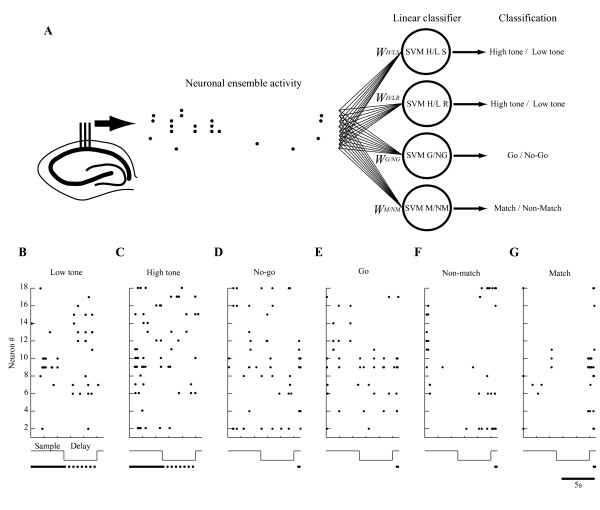
**Decoding stimulus perception, stimulus retention, motor selection, and stimulus comparison from hippocampal ensemble activity**. *A*: The decoder classifies binary labels--low and high tones during sample and delay periods, no-go and go responses, and match and non-match tones during test periods--based on neuronal ensemble activity patterns. The dots show the raw spiking activity pattern of the hippocampal ensemble obtained while the rat performs the DNMS task. The circles are linear classifiers, each of which linearly combines the spiking activities as inputs (weighted sum plus bias; bias component not shown). The weights are determined using a statistical learning algorithm (linear support vector machine (SVM)) applied to each training dataset such as those shown in *B-G*. Finally, each SVM specialized for stimulus perception (SVM H/L P), stimulus retention (SVM H/L R), motor selection (SVM G/NG), and stimulus comparison (SVM M/NM), classifies the behavioral events. *B-G*: Typical raster plots of raw spiking activity of hippocampal ensemble #1 used for SVM classification in low tone, high tone, no-go response, go response, non-match, and match trials, respectively. The middle traces show sequences of events within a trial. The lower lines show intervals in which SVM used ensemble activity to classify low and high tones during the sample (solid lines in B and C) and delay (dotted lines in B and C) periods, or no-go and go responses or non-match and match tones during test (solid lines in D-G) periods.

**Figure 2:**
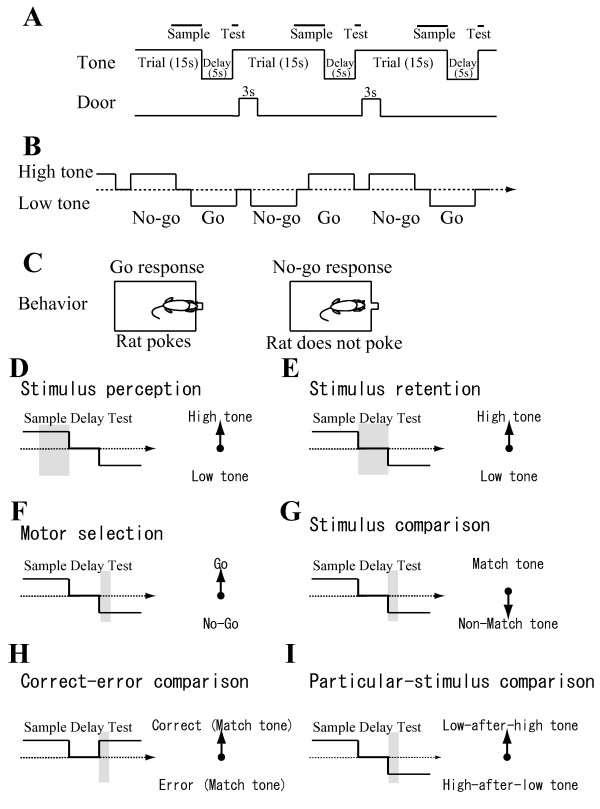
**Auditory-guided continuous delayed non-matching-to-sample task**. *A*: Sequence of events within a single continuous trial and delay. Each trial consisted of a 15 s tone presentation and a 3 s response-opportunity period following a 5 s delay period. The response-opportunity period started 1 s after the onset of the tone presentation of each trial. The upper bold lines represent sample and test periods. *B*: Typical sequence of trials, delays and responses within a session. *C*: In a trial in which a non-match tone is presented, the tone differs from that presented in the previous trial; rats poke their nose into a hole to obtain a reward. In a trial in which a match tone is presented, the same tone is repeated; rats do not poke their nose into the hole. Even when rats poke their nose on match trials, they receive no reward. Consequently, rats learn not to poke their nose in match trials. *D-I*: Examples of a sequence with sample, delay, and test periods for stimulus perception (D), stimulus retention (E), motor selection (F), stimulus comparison (G), correct-error comparison (H), and particular-stimulus comparison (I). Each shaded box shows the interval for the decoder. Each arrow on the right side presents an example vector to one of the labels being classified.

We specifically examined the activities of the neuronal ensembles to provide quantitative constraints for hippocampal function. We used a decoding technique based on a linear classifier for neuronal ensembles (Figure 1). The decoding approach consists of training and regularizing a classifier to learn the map from neuronal ensemble activity to each behavioral label (Figures 2D-2I) (see *Methods*), as has been done similarly in recent studies of the inferior temporal and motor cortices [18-20]. The classifier learns the map directly from the training sets and generalizes it to a novel ensemble activity instead of using prior knowledge of the probability distribution of the training sets. The input comprises neuronal ensemble activities from simultaneously monitored cells such as those shown in Figures 1 and 4. After training a binary classifier using a leave-one-out cross-validation method, the classifier is useful to decode the ensemble activity in a novel trial of tasks. Using such classifiers that can be implemented easily in neuronal networks of the hippocampal CA1, we can assess the lower bound on the information available in the ensemble activity in a single trial [19].

### Classification performance in neuronal ensembles and combination

**Figure 3:**
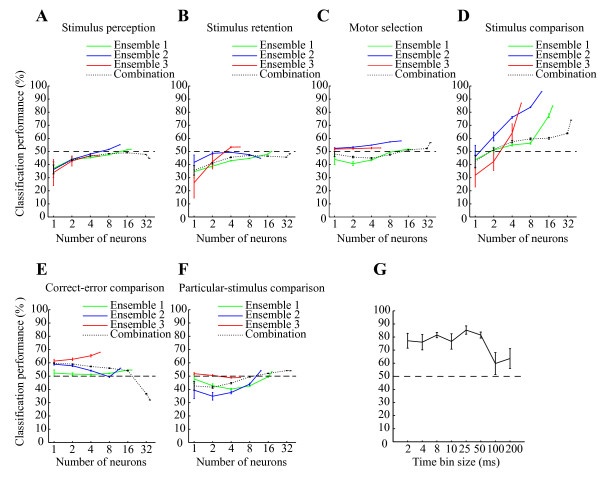
**Classification performances and time resolution of code for stimulus comparison**. *A-F*: Classification performance for linear classifier on test data (not used for training) as a function of the number of neurons on a logarithmic scale in ensembles #1 (green), #2 (blue), and #3 (red) and combination (neurons pooled over all ensembles; dotted black) for decoding stimulus perception (A), stimulus retention (B), motor selection (C), stimulus comparison (D), correct-error comparison (E) and particular-stimulus comparison (F). The input from each neuron was the spike count in consecutive 25 ms bins. The intervals are set at -10 - -5 s (A), -5 - 0 s (B) and 0 - 1 s (C-F) after the test tone onset. *G*: Time resolution of code for stimulus comparison. Average classification performance over three ensembles as a function of bin size (2-200 ms, temporal resolution) to count spikes within a 0-1 s window after the test tone onset for stimulus comparison. Error bars show the SEM. Dashed lines show chance levels (= 50%).

**Figure 4:**
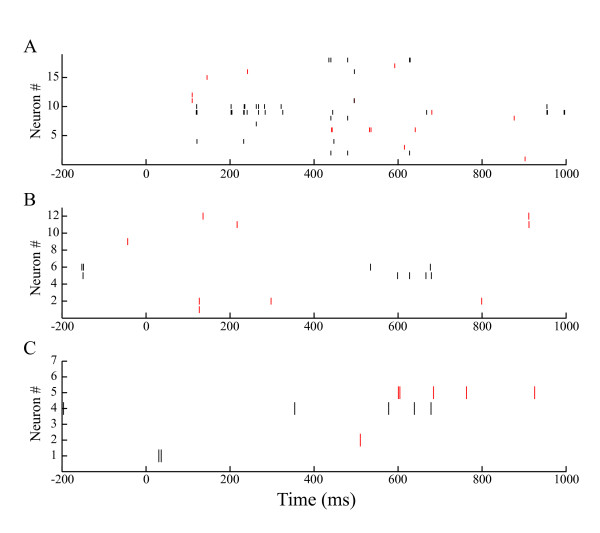
**Raster plots of hippocampal activity**. Typical raster plots of raw spiking activity of hippocampal ensembles #1 (**A**), #2 (**B**), and #3 (**C**) on match (red) and non-match (black) trials during the test period (0-1,000 ms) in a single session. Red and black bars respectively show spikes on match and non-match trials.

**Figure 5:**
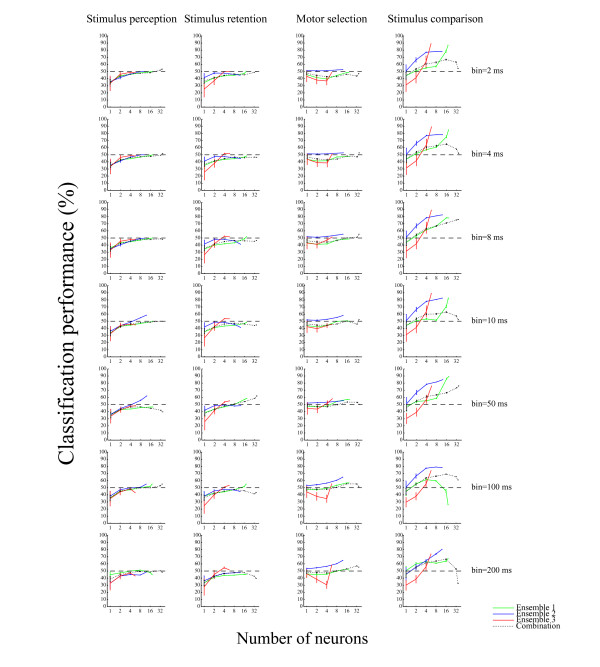
**Classification performance over various time intervals**. Classification performance for a linear classifier on test data as a function of the number of neurons in ensembles #1 (green), #2 (blue), and #3 (red) and combination (neurons pooled over all ensembles; dotted black). Each column shows roles of stimulus perception, stimulus retention, motor selection, and stimulus comparison. Each row shows performances over 2 ms, 4 ms, 8 ms, 10 ms, 50 ms, 100 ms, and 200 ms time intervals on a logarithmic scale. Error bars show the SEM. Dashed lines show chance levels (= 50%).

We used the classifier approach to determine the functional roles of the ensembles of hippocampal CA1 during the DNMS task. Figure 3 presents the cross-validated performance of the classifier for each neuronal ensemble. The spiking activity of the ensembles was sufficient to classify a match tone and non-match tone (stimulus comparison) with maximum accuracy of 96% over a 25 ms time interval (Ensemble #1: 84.8%, *P *< 10^-6^; Ensemble #2: 95.7%, *P *< 10^-12^; Ensemble #3: 87.0%, *P *< 10^-7^; chance = 50%; binomial test; see *Methods*; Figures 3D and 4). Similarly, we determined the functional roles of the hippocampal ensembles in classifying go and non-go responses (motor selection) and high and low tones during sample and delay periods (stimulus perception and stimulus retention). The hippocampal ensembles were not capable of classifying these roles with sufficient accuracy (For sensory perception: Ensemble #1: 52.0%, *P *> 0.3; Ensemble #2: 55.2%, *P *> 0.1; Ensemble #3: 46.6%, *P *> 0.6; For stimulus retention: Ensemble #1: 50.0%, *P *> 0.4; Ensemble #2: 44.8%, *P *> 0.7; Ensemble #3: 53.4%, *P *> 0.2; For motor selection: Ensemble #1: 51.3%, *P *> 0.3; Ensemble #2: 58.1%, *P *> 0.05; Ensemble #3: 52.7%, *P *> 0.2; chance = 50%; binomial test; see *Methods*; Figures 3A-C). Moreover, we compared correct and erroneous responses during the test periods (correct-error comparison), and low-test tone periods preceded by high sample tones (low-after-high) and high test tone periods preceded by low sample tones (high-after-low) (particular-stimulus comparison). The results show that the hippocampal ensembles were also not capable of classifying these with significant accuracy (For correct-error comparison: Ensemble #1: 54.5%, *P *> 0.2; Ensemble #2: 56.0%, *P *> 0.2; Ensemble #3: 68.0%, *P *> 0.02; For particular-stimulus comparison: Ensemble #1: 53.3%, *P *> 0.2; Ensemble #2: 54.1%, *P *> 0.2; Ensemble #3: 48.9%, *P *> 0.5; chance = 50%; binomial test; see *Methods*; Figures 3E and 3F). The performance values depicted in Figure 3 portray how accurately downstream neurons were able to classify the functional roles in a single trial, as determined using the computation of a weighted sum of spikes over a 25 ms time interval. On the other hand, the classifier performance of the neuronal ensembles, as well as that of the combination (neurons pooled over all three ensembles), was enhanced approximately linearly with the logarithm of the number of participating cells (Figure 3D), indicating that the codes for the stimulus comparison are distributed across cells, unlike that described by the grandmother cell doctrine. Moreover, irrespective of the large number of cells in the combination, the accuracy of the combination was markedly lower than those of the ensembles over all the other examined time intervals (e.g., combination of 36 cells, 73.9%; Ensemble #2 of 12 cells, 95.7% over a 25 ms time interval; Figures 3D and 5).

### Time resolution of code for stimulus comparison

By investigating the degree to which classification performance depended on the bin size of the spike count, we examined the temporal resolution of the ensemble code. Bin sizes of 2-50 ms yielded better performance than larger bin sizes (Figure 3G). A 2-ms bin typically contained zero or one spike. Consequently a few spikes from a small number of neurons are sufficient to encode the stimulus comparison in the hippocampal ensembles.

## Discussion

In this study, the activity of small neuronal ensembles (6-18 cells) in the hippocampal CA1 was used to classify the stimulus comparison with good accuracy over brief time intervals (2-50 ms). They were not useful to classify the stimulus perception, stimulus retention, or motor selection. The accuracy of the ensembles for the stimulus comparison was markedly higher than that of the combination (36 cells).

### Small neuronal ensembles in the hippocampal CA1 specifically code stimulus comparison

During test periods in which the rats prepare for go/no-go responses, we examined the stimulus comparison as well as the motor selection. The motor selection of go/no-go responses contains the stimulus comparison of match/non-match tones because, in correct trials, go and no-go responses respectively correspond to non-match and match tones. It is possible that the low accuracy of the ensembles for the motor selection suggest that the activities of the neuronal ensembles are not useful to classify the stimulus comparison. To verify the stimulus comparison without the influence of the motor functions, we examined ensemble activities during match and non-match tones to which identical behavioral responses (go responses) were conducted. Moreover, to exclude the influences of correct and erroneous responses and the particular stimuli being presented, we examined the classification performance of the ensemble activity in relation to the correct-error difference and the particular-stimulus presentation. The results show that, using a linear classifier that can be realized easily in downstream neurons by summating appropriately weighted inputs, we can clearly characterize that at the ensemble level, the most available information in the hippocampal CA1 in each single trial is specifically the stimulus comparison: it is neither the correct-error difference nor the particular-stimulus presentation.

Based on the results of ensemble analyses on a trial-to-trial basis, Deadwyler and colleagues inferred multiple representation and conjunctive encoding in activities of small hippocampal ensembles consisting of 10 neurons [7]. In contrast with the present results, they showed that the stimulus comparison in the hippocampal ensembles was highly correlated with correct and erroneous responses. The reason for the inconsistent results might be differences in the tasks (spatial vs non-spatial versions of DNMS task) and differences in the ensemble analysis tools (canonical discriminant analysis vs. SVM). Furthermore, because we examined neuronal ensemble activities only in the hippocampal CA1, the activity of the hippocampal CA3 analyzed in the Deadwyler's study might explain the inconsistency of the results. The results of the present study illustrate that small neuronal ensembles in the hippocampal CA1 are specifically dedicated to stimulus comparison. The results support the notion that the hippocampus contributes to memory by identifying consistencies across experiences that constitute new associations, as reported from some previous studies [4,7,10,15].

### The classification performance for the stimulus comparison of neuronal ensembles is better than that of their combination

Several lines of evidence [21-24] suggested that firing rate modulations of individual neurons and spike timing among neurons contain information in the neuronal ensembles. Given that the small neuronal ensembles in the hippocampal CA1 contains information related only to the firing rate modulations of individual neurons, the classification performance of the combination of all neurons in all the ensembles recorded from different sessions is expected to be the sum of the performance of the ensembles. However, in the present study, almost all classification performances of small neuronal ensembles in the hippocampal CA1 for the stimulus comparison were higher than that of the combination. Moreover, the ensembles yielded better performance in a 2-50 ms window. From this perspective, we presume that the timing of spikes of 2-50 ms resolution in the ensembles recorded in the identical session, such as second or higher order synchronies, contain much information for the stimulus comparison in the hippocampal CA1 at the ensemble level.

### Information of the stimulus comparison may be distributed throughout the hippocampal CA1

To elucidate the decoding ability of small neuronal ensembles in the hippocampal CA1, we examined the spatiotemporal firing patterns of small neuronal ensembles consisting of a maximum of 18 cells. Results of a previous study suggested that each of the discreetly located clusters of neurons is dedicated to a different aspect of the spatial version of the DNMS task [25]. Therefore, not all represented information in the hippocampus might be induced from activity of the small neuronal ensembles detected in the present study. That viewpoint, together with the results of analyses of firing rates of individual single cells [7-10], suggests that larger neuronal ensembles in the hippocampus are associated with all examined events in addition to the stimulus comparison in the present study. Nevertheless, our results demonstrate that, in spite of using randomly selected neurons, all small neuronal ensembles in all the events we examined specifically classified the stimulus comparison. For that reason, we infer that the information of stimulus comparison is not restricted, but is instead distributed throughout the hippocampal CA1. It can be retrieved robustly by the ensemble activity of any subcombination in it.

On the other hand, in this study, the SVM classifiers behave as the target of the hippocampal CA1, such as the entorhinal cortex and subiculum. Those targets receive spikes not from the entire hippocampal CA1 but from a subset of it [26]. Consequently, the activity of a small neuronal ensemble in the hippocampal CA1 over brief time intervals during a single trial demonstrates the possibility that the targets of hippocampal CA1 work to perform, accurately, tasks that requires a stimulus comparison, as described in a recent study [27].

## Conclusion

The results show that a neuronal ensemble in the hippocampal CA1 acts as a comparator during a recognition memory task. Our approaches and findings revealed the cognitive functioning of the hippocampal CA1 from the neuronal ensemble activity in a single trial, supporting the development of new solutions for reading cognitive functions from the brain such as brain-machine interfaces [18,28]. Further assessment of the application of neuronal ensemble activity in the cognitive version of brain-machine interfaces can be achieved through experiments incorporating the sensory feedback of brain-controlled actuators in real-time.

## Methods

### Task procedure

Three male Wistar rats were extensively handled; then food was deprived to approximately 80-90% of their ad libitum body weight. Lights were left on in the colony room between 8 A.M. and 9 P.M Experiments were conducted between 9 A.M. and 6 P.M. The rats were trained to perform an auditory-guided continuous DNMS task [8,9]. A training session consisted of approximately 200 trials performed for approximately 1 h. The criterion for performance was 80% correct trials per session. In each trial (Figure 2A), one of two tones (high tone: 10 kHz, 85 dB SPL; low tone: 2 kHz, 85 dB SPL) was randomly presented for 15 s following a 5 s delay period. One second after the tone onset, a guillotine door was opened for 3 s to show an illuminated response panel immediately behind the door. Trials were continuously performed with intervening delays (Figure 2B). A food reward was delivered immediately after the go response in a non-match trial, in which the presented tone (high/low) differed from that in the preceding trial. During each trial, a rat was required to make a go response in non-match trials and a no-go response in match trials (Figure 2C). During the delay period, the rat had to remember which stimulus (high/low tones) had been presented most recently. This task design enabled us to dissociate hippocampal neuronal activities associated with a) stimulus perception (difference in activity during sample periods (Figure 2A) between high and low tones) (Figure 2D), b) stimulus retention (difference in activity during delay periods (Figure 2A) between preceding high and low tones) (Figure 2E), c) motor selection (difference in activity during test periods (Figure 2A) between go and no-go responses) (Figure 2F), d) stimulus comparison (difference in activity during test periods between match tone (erroneous go response) and non-match tone (correct go response)) (Figure 2G), e) correct-error comparison (difference in activity during test periods between correct and erroneous responses when match tones was presented) (Figure 2H), and f) particular-stimulus comparison (difference in activity between low test tone periods preceded by high sample tones (low-after-high) and high test tone periods preceded by low sample tones (high-after-low)) (Figure 2I). All behavioral events were controlled using custom-written software running with another software program (Labview; National Instruments Corp., Austin, TX).

### Animal preparation and recordings

**Figure 6:**
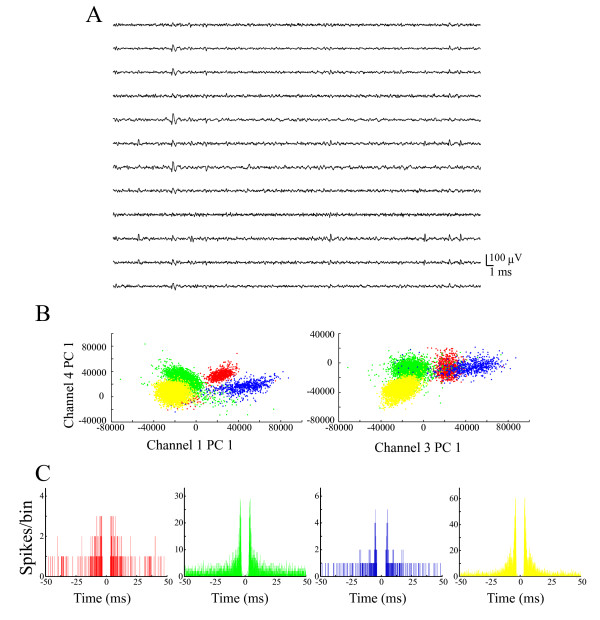
**Example of single units sorted by ICSort with dodecatrode**. (A) Raw signals from one dodecatrode. (B) Scatter plots on two different feature spaces for the sample dataset. The sorted four units (clusters) are shown in different colors. (PC, principal component). (C) Corresponding autocorrelation functions with window of ± 50 ms. The bin size is 0.1 ms.

The respective activities of three neuronal ensembles (Ensemble #1, 18 cells from rat #1; Ensemble #2, 12 cells from rat #2; Ensemble #3, 6 cells from rat #3) were recorded from the hippocampal CA1 (3-4 mm posterior to the bregma, 1.5-3.5 mm from the midline) of three rats performing the DNMS task using multi-neuronal recording with 12-channel electrodes (dodecatrodes) [29-33]. All experimental procedures were performed in accordance with NIH and Kyoto University guidelines and were conducted with the approval of the Animal Research Committee, Kyoto University. We recorded neuronal data only when we had confirmed that the distributions of spike amplitude across channels were constant. Multi-neuronal activities were amplified, filtered (band-pass frequency range, 500 Hz-10 kHz) and recorded at 20 kHz on a custom-made PC with three 24-channel A/D converters (16-bit resolution; Contec Co. Ltd., Osaka, Japan) (Figure 6A). After our unique spike sorting (ICSort) [29-33] (Figures 6B and 6C), we identified pyramidal cells based on their wide spike shape (mean width (peak-to-peak time): > 0.2 ms), low average firing rate (< 5 Hz). and a sign of bursts in the auto-correlogram [34,35]. We used spike trains only from putative pyramidal neurons that showed clear refractory periods (1-2 ms) (Figure 6C), high signal-to-noise ratios (> five times the noise level) (Figure 6A), and sufficiently high firing rates (> 0.1 Hz) [29-33]. Additionally, we considered only units with a high isolation quality index (isolation distance > 20) [36] because units having a poor isolation quality engender false conclusions [37].

### Neuronal ensemble analysis

We explored codes by counting spikes in successive bins of size *w *within the interval starting *i*_*s *_s after the test-tone onset and ending *i*_*e *_s after the test-tone onset. We describe the results for different values of these parameters. The default condition was *w *= 25 ms. Parameter *w *controls the time resolution of the code: we used *w *= 2 ms, 4 ms, 8 ms, 10 ms, 25 ms, 50 ms, 100 ms, and 200 ms. The interval parameters were set as *i*_*s *_= -10 and *i*_*e *_= -5 for the stimulus perception because, during this period, a sensory stimulus (high/low tone) is obtained and a rat does not make a go/no-go response. The parameters were *i*_*s *_= -5 and *i*_*e *_= 0 for the stimulus retention because, during this period, a sensory stimulus (high/low tone) is not obtained and must be retained in memory. The parameters were *i*_*s *_= 0 and *i*_*e *_= 1 for the motor selection because, during this period, a rat prepares a motor response (go/no-go response). The parameters were *i*_*s *_= 0 and *i*_*e *_= 1 for the stimulus comparison, correct-error comparison, and particular-stimulus comparison because, during this period, a rat compares a sample tone with a test tone before it makes a go/no-go response.

Let *s*(*i*_*s*_, *w, n*) denote the number of spikes in the interval between *i*_*s *_+ *nw *ms and *i*_*s *_+ *(n+1)w *ms, where *n *denotes the number of bins in the vector and *i *is an integer such that the entire interval extends from *i*_*s *_ms to *i*_*e *_ms. The single-cell activity *t *is defined as



This vector was used as the input to the decoding classifier. When considering the activity of multiple neurons, we concatenated the corresponding activity vectors and used the concatenated vector as the input to the classifier. The dimensionality of the input is therefore (*n*+1)*N*, where *N *represents the number of neurons in an ensemble or a combination. For a neuronal combination (neurons pooled over all three ensembles), this concatenation step assumes independence among different neurons. For a neuronal ensemble, correlations between simultaneously monitored neurons in an ensemble must contain additional information and must reveal additional aspects of the neural codes.

We used a leave-one-out cross-validation method for training and testing the data. Data were always divided in all cases into a training set and a test set. The training set comprised *T-1 *trials of each event, whereas the test set included the remaining one trial; *T *represents the number of trials in one session. Therefore, we used *T *pairs of training and test sets.

We specifically investigated six functional roles: stimulus perception, stimulus retention, motor selection, stimulus comparison, correct-error comparison and particular-stimulus comparison. For stimulus perception, the labels represent which tones were presented during the sample periods (high or low tone) (Figure 2D). For stimulus retention, the labels denote which tones were presented during the delay periods (high or low tone) (Figure 2E). In correct trials, the motor selection of go/no-go responses can be examined. For motor selection, the labels denote which behavioral responses a rat made during the test periods in correct trials (go or no-go response) (Figure 2F). The go responses can be divided into correct and erroneous go responses. Comparing activities between correct go responses during non-match tones and erroneous go responses during match tones, the stimulus comparison: match tone vs. non-match tone on the identical behavior (go response) can be examined. For stimulus comparison, the labels denote whether the sample tone and test tone differed in trials in which a rat made go responses during the test periods (match tone (erroneous go response) or non-match tone (correct go response)) (Figure 2G). For correct-error comparison, the labels denote whether a rat made correct or erroneous responses during the test periods (correct no-go response or erroneous go response during match tones) (Figure 2H). For particular-stimulus comparison, the labels show which pair of non-match tones was presented during the test periods (high-after-low or low-after-high tone) (Figure 2I). For all three rats examined, the number of trials in which the rats made erroneous no-go responses during non-match tones (< 3) was insufficient for statistical tests and analyses; we did not examine binary labels that include an erroneous no-go response during non-match tones for the stimulus comparison and correct-error comparison.

We trained one binary classifier for each role. The classification performance, as portrayed in all plots, represents the share of correct decoding for test data (i.e., data not used by the classifier during training).

We compared the performance of different statistical classifiers including Perceptron, Fisher's linear discriminant classifier, and Support Vector Machine (SVM) [38] with a linear kernel using sample ensemble activity. The SVM classifiers yielded the best performance. In addition, the SVM architecture can be easily realized in the hippocampal as a threshold sum of weighted synaptic inputs. Therefore, we used SVM implemented in the software package '***libsvm' ***.

The linear classifier is expressed as y = sign [g(***t***)]. The calculation of g(***t***) is a linear function of the form of , where *t*_*i *_is single-cell activity, *w*_*i *_is the weight of *i-*th cell, and ***b ***is the bias. In fact, SVM finds the optimal weights and bias. We initially tested the performance of the classifier on a small subsample of the data, exploring a large set of parameters. Then we used the optimized parameters for analysis of the complete dataset. The parameters for ***libsvm ***were *C *= 10, *S *= 0, and *T *= 0.

The graphs presented in the text show that the *N *neurons used as the input to the classifier were all possible combinations of *N *neurons if the number of all possible combinations was < 1,000 or were 1,000 combinations chosen randomly from among all possible combinations of *N *neurons. We report the average obtained from all random neuronal subensembles and subcombinations.

### Test of significance for decoding performance

We used the binomial test to determine the statistical significance of decoding performance. The probability of predicting *X *times the correct labels in *N *trials by chance is given as a binomial distribution:



Using this binomial distribution, we were able to establish the statistical significance of the observed values for the decoding performance.

All analyses were performed using custom-written software (C++) and another software program (MATLAB; The MathWorks Inc., Natick, MA).

## Authors' contributions

ST and YS designed the experiments. ST performed the experiments. ST analyzed the data. ST and YS wrote the manuscript.
